# Neurosurgery aspirants in UK medical schools: a national cross-sectional analysis of demographics, motivations, and confidence (FAST study)

**DOI:** 10.1186/s12909-026-08845-0

**Published:** 2026-03-13

**Authors:** Tomas Ferreira, Alexander M. Collins, Oliver Feng, the FAST Collaborative, Megan Fallows, Ioan Valnarov-Boulter, Hsuan-Tung Kuo, Aryan A Sagdeo, Bethany McDermott, Michael Y Luo, Anson Wong, Erin Fitzsimons-West, Sharon Yuen Shan Ho, Lubana Hemayet, Meghna Sreekumar, Samantha Burley, Andreas Stavrinou, Heather A Lewis, Hugo Bernie, Olivia Rowe, Catherine L Otoibhi, Dora Pascoe, Leya Luhar, Rakeem Khalid Basith, Mayisha Samiha, Tushar Rakhecha, Mia Mäntylä, Molly Doyle, Chiamaka Anthony-Okeke, Basma R Khan, Safa Razzaq, Malvi Shah, Ryan White, Brandon S H Low, Olivia King, Flora E MacInnes, Yasmin Al-Rawi, Hannah Layton-Joyce, Manuel Giardino, Maisha Hayat, Anjali Cyril, Rivya Mathews, Ashleigh Stirling, Adam Mohamed Capdevila, Laura AE Munn, Sara Ali, Zulaikha Bibi, Mackenzie F Garlick, Lily Chadwick, Bilal Qureshi, Fariha Hasan, Zoe Mary Constantinou, Beray Berkay, Daniel C Chalk, Joseph Nicholson, Sakshi Garg, Jessica Sinyor, Louis R Dowland, Alfaiya Hashmi, Freya F Semple, Tan Jit Yih, Dev Ranka, Kajoke M S Avolonto, Elliot Skittrall, Madison Gill, Ben Sweeney, Ria Bhatt, Humaira Khanom, Jonathan Craven, Harini Elankhumaren, Hannah Glover-Adams, Ishagit Kaur, Maia C Letts, Krishnika Vetrivel, Hesham Zalghana, Ananya Jain, Anna Collins, Sophie West, Muhammed Asif, Yasmin Owadally, Urja Ashish Mhatre, Nikita Sanctis, Ahamed Hafeezul Nashith Ahamed Rizwaan, Carys M. Francis, Rida Khan, Vaishvi Dalal, Sundaramoorthy Balasubramanian, Ayeza Akhtar, Sudhanvita Arun, Saimathusan Sivakandarajah, Sourab Chand Surana, Rosemary M Davis, Daria Maria Bageac, Ibrahem Al-Obaidi, Daniel Magee, Ali Hamed Mahmood Al-Nakeeb, Katie Appleton, Amelia Dickson, Subham Roy, Mohammed Suhaib Amin, Sakshi Roy, Tessa Yau, Pakhi Goel, Abderrahmane El Guernaoui, David Gringras, Soo Sun Chong, Sara Kidher, Aaliyah J Bax, Rachelle Thevathas, Ajwa Fayaz, Cameron Thurlow, Vera Onongaya, Pranjil Pokharel, Shama Maliha, Phoebe Hatrick, Rhiannon Tanner, India R Barrons, Louis Naraine, Artemis Prevot, Muaawiyah Shaheen, Farah Ahmed, Mathumetha Rubaratnam, Michael E Bryan, Harsh Sai Modalavalasa, Sushmhitah Sandanatavan, Aleksandra Dunin-Borkowska, Kavyesh Vivek, Anhukrisha Karthikeyan, James Brawn, Sophie Kidd, Jack M Read, Saarah Saanan Khan, Gregory A Rowland, Tawfique Rizwan, Khyatee Shah, Alexa McCloskey, Nidhi Sachidananda, Lydia Melaku, Vyom Patel, Alyssa Weissman, Iliyah Shahbeik, Maeve K Mulchrone, Medha Pillaai, Emily R Finbow, Jessica Lo, Jui Kanetkar, Hannah Bolton, Timo L Kuerten, Leah Njenje, Matilda Gardener, Emily Carver, Mairi C Docherty, Nabilah Ali, Shereen Ahmad, Shiksha Guru, Luke Dcaccia, Guilherme Movio, Anushka Pujari, Sydney LA Barnes, Tommy Wai Kei LI, Khadijah Khan, Riya Patel, Momina Iqbal, Samuel Foxcroft, Sewa A Badejo, Chloe Milton, Ellena K Briggs, Raghad A F Sallama, Emma Marsh, Muhammad Hamza Shah, James A Cairn

**Affiliations:** 1 Bristol Medical School, University of Bristol, Bristol, UK; 2 St Thomas’ Hospital, Guy’s & St Thomas’ Trust, London, UK; 3 London School of Economics and Political Science, London, UK

**Keywords:** Neurosurgery, Career intentions, Medical students, Workforce planning, FAST study, Specialty preferences

## Abstract

**Background:**

Neurosurgery is among the most competitive specialties in the UK, yet national data on who aspires to it and why are limited. Using the FAST study, we compared medical students who selected neurosurgery with peers choosing other specialties, examining demographics, extracurricular activity, certainty, confidence, and knowledge of the training pathway.

**Methods:**

Secondary analysis of the FAST cross-sectional survey of UK medical students conducted December 2023 to March 2024. Responses were collected via an online questionnaire covering demographics, education, extracurricular activity, certainty, confidence, knowledge of training pathways, and factors influencing specialty choice. We compared neurosurgery aspirants with the remaining cohort using descriptive statistics and logistic regression to estimate odds ratios with 95% confidence intervals. Bonferroni corrections were applied where appropriate.

**Results:**

Of 8,395 respondents, 212 students selected neurosurgery as their preferred specialty (2.53%). Interest declined sharply with seniority, from 4.5% of first-year students to 0.6% of final-year students. Compared with the national cohort, aspirants were more often male and from non-White ethnic groups. Private schooling was more frequent 29.7% vs 26.0% but not significant. Aspirants reported greater certainty about career choice (OR 2.43, *p* < 0.0001) and higher self-reported knowledge of the neurosurgery pathway. Confidence in securing a training post was low (20.8% confident) and the odds of low confidence were higher than the national cohort adjusted OR 1.37, *p* = 0.04. Males reported higher knowledge 67.3% vs 42.7% in females and greater confidence. Factors more strongly associated with choosing neurosurgery included intellectual challenge (OR 2.56, *p* < 0.0001), research opportunities (OR 4.07, *p* < 0.0001), and interest in specific conditions (OR 3.12, *p* < 0.0001). Lifestyle considerations were less influential than in peers: work-life balance (OR 0.33, *p* < 0.0001), compatibility with family life (OR 0.37, *p* < 0.0001), and job stress (OR 0.41, *p* < 0.0001).

**Conclusion:**

Despite high levels of certainty and pathway knowledge, neurosurgery aspirants reported low confidence in securing a training post, with marked gender and socioeconomic disparities. Interest declines steeply with seniority, suggesting that early enthusiasm often fades due to limited exposure and perceived inaccessibility. Targeted early interventions, including early and sustained exposure, and visible mentorship, may help ensure informed, sustained interest among those best suited to the field.

**Supplementary Information:**

The online version contains supplementary material available at 10.1186/s12909-026-08845-0.

## Background

Neurosurgery is one of the most competitive medical specialties to which to gain entry in the United Kingdom. In 2024, 354 applicants competed for only 18 training posts, illustrating the intense demand and limited opportunities within the field [1, 2]. Furthermore, neurosurgical training is characterised by significant challenges, including its long duration, demanding workload, and high risk of burnout, which collectively present substantial barriers to trainees [3–7].

Despite the specialty’s apparent popularity, there is a paucity of data on the demographic and socioeconomic characteristics of UK medical students aspiring to this career. Similarly, little is known about their confidence, certainty, or preparedness regarding the neurosurgical training pathway. Neurosurgery offers intellectual challenges, diverse clinical experiences, and research opportunities. However, limited exposure to the field during medical school, partly due to an increasing emphasis on general practice principles, has left it underrepresented in the curriculum [8, 9]. This lack of exposure can delay or deter students from pursuing neurosurgery, underscoring the importance of early engagement [10]. While existing studies suggest that early exposure to neurosurgery, access to mentors, and targeted educational initiatives can improve student interest and confidence in the specialty [11–15], much of this evidence has been derived from targeted cohorts, such as those attending neurosurgical conferences, which is likely to introduce bias. To date, no large-scale, nationally representative study has characterised medical students aspiring to neurosurgery, leaving critical gaps in our understanding of this group. Addressing these shortcomings is critical for workforce planning, particularly to tackle inequities in recruitment and reduce attrition among doctors.

The Factors Affecting Specialty Training preference (FAST) study, the largest cross-sectional survey of UK medical students, investigated specialty preferences and the factors influencing these decisions [16–18]. This analysis of FAST data focuses on neurosurgery aspirants within the study population, evaluating their demographic profiles, socioeconomic backgrounds, and extracurricular achievements, offering the first nationally representative characterisation of this group. We aim to compare neurosurgery aspirants with the broader medical student population and identify the key factors driving their interest in the specialty. These insights may have implications for optimising selection processes, addressing diversity challenges, and supporting equitable access to neurosurgical training opportunities.

## Methods

This investigation was conducted using FAST study data. The full methodology of the FAST study has been previously described in detail [16–18] and is summarised here with modifications specific to this analysis.

### Study design

The FAST study was a national, multi-centre, cross-sectional survey of UK medical students. Here, we focus on participants who selected neurosurgery as their preferred specialty. Data were collected using an online questionnaire hosted on the GDPR-compliant Qualtrics platform (Provo, Utah, USA). The survey contained 17 questions, utilising Likert scales, multiple-choice questions, and free-text entries to capture detailed insights into participants’ demographic profiles, socioeconomic backgrounds, and factors influencing specialty preferences. The survey can be found in Supplementary Materials.

Data for the overarching FAST study were collected between December 4, 2023, and March 1, 2024. Responses relevant to neurosurgery aspirants were extracted from this dataset for this analysis.

### Survey content

The survey comprised three sections. Section 1 collected demographic data and participant consent. Section 2 focused on specialty choice, knowledge, and guidance received regarding training pathways. Section 3 asked participants to rate the importance of various factors influencing specialty preferences and included a free-text option for additional input. This analysis specifically examined responses related to neurosurgery as a career choice.

Socioeconomic background was assessed using self-reported school type, which is commonly used in UK educational research; other measures such as POLAR quintile, IMD, or parental occupation were not collected within the FAST survey.

### Participant recruitment and eligibility

As previously described [16–18], the survey was distributed through a national network of FAST collaborators, representing all eligible UK medical schools across all devolved nations. Recruitment strategies included social media outreach, email distribution lists, and medical student societies. All current students enrolled at GMC-approved UK medical schools were eligible to participate. Responses from individuals indicating neurosurgery as their intended specialty were included in this separate analysis. Response rates varied per institution and can be found in the Supplement.

### Data processing and storage

Responses were anonymised and restricted to institutional email addresses to ensure data integrity and mitigate duplication. Data were stored securely in a password-protected Microsoft Excel file, accessible only to the central study team.

### Data analysis

Descriptive and inferential statistical analyses were performed using Microsoft Excel (V.16.71), and R (V.4.4.1). Multivariable logistic regression models were fitted to explore relationships between demographic characteristics and neurosurgery-specific preferences, using odds ratios (ORs) with 95% confidence intervals (CIs) and *p*-values (significance threshold *p* < 0.05, with Bonferroni corrections as appropriate). In all models, the generalised variance inflation factor for every predictor (adjusting for the number of factor levels) was at most 1.3, indicating no significant multicollinearity.

### Ethical considerations

Per UK NHS Health Research Authority guidance, formal ethical approval was not required for this investigation, as the study involved anonymous, non-interventional survey data. Explicit informed consent was obtained via the survey platform prior to participation, and all respondents were provided with a Participant Information Sheet outlining the study’s purpose, voluntary nature, and data confidentiality. The first survey page included a mandatory consent question required for progression, and participants were informed that they could withdraw their data at any time by contacting the study lead. All procedures complied with the principles of the Declaration of Helsinki.

## Results

### Demographics

Of 8,395 participants, 212 (2.55%) identified neurosurgery as their preferred specialty, with 199 of these provided all requested demographic information. The remaining 13 withheld their schooling (*n* = 12) and/or gender (*n* = 3), and were excluded from the regression analysis. The median age of neurosurgery aspirants was 21 years (IQR 19–22), similar to the national cohort’s median of 22 years (IQR 20–23). Gender distribution among neurosurgery aspirants consisted of 51.9% female and 46.2% male, in contrast to the overall study population, where females constituted 68.2% and males 30.2%. Notably, neurosurgery was selected by 3.9% of male participants compared to 1.9% of females, indicating a disproportionate representation of males in the neurosurgery cohort relative to the national sample.

Neurosurgery aspirants were more likely to have attended private schools (29.7%) compared to the national cohort (26.0%), though this difference did not reach statistical significance (*p* = 0.132). Differences were observed between ethnicities, with White students comprising 32.1% of neurosurgery aspirants compared to 53.3% nationally. All other ethnic groups were proportionately overrepresented among neurosurgery aspirants. Conversely, students with a parent or sibling in medicine were underrepresented, comprising 17.0% of the neurosurgery-inclined cohort versus 20.5% nationally (Table 1).

**Table 1:** Demographic characteristics of neurosurgery aspirants versus the national cohort

Characteristic	Neurosurgery Cohort	National Cohort
	Percentage (n)	Percentage (n)
Ethnicity		
Asian or Asian British	42.9% (91)	30.2% (2,532)
Black, Black British, Caribbean or African	7.5% (16)	5.8% (4,91)
Mixed or multiple ethnic groups	6.6% (14)	5.3% (4,47)
White	32.1% (68)	53.3% (4,474)
Other	9.4% (20)	4.2% (3,54)
Prefer not to say	1.4% (3)	1.2% (97)
Gender		
Female	51.9% (110)	68.2% (5,727)
Male	46.2% (98)	30.2% (2,537)
Non-binary	0.5% (1)	0.9% (78)
Prefer not to say	1.4% (3)	0.6% (53)
Course type		
Postgraduate	12.7% (27)	17.9% (1,505)
Undergraduate	87.3% (185)	82.1% (6,890)
Previous schooling		
Comprehensive state school	40.1% (85)	48.4% (4,061)
Selective state school or grammar school	29.7% (63)	22.4% (1,883)
Private school (fee-paying)	24.5% (52)	26.0% (2,180)
Prefer not to say	5.7% (12)	3.2% (271)
Fee status		
Home	71.7% (152)	87.0% (7,305)
EU/EEA	5.7% (12)	3.5% (293)
International (non-EU)	22.6% (48)	9.5% (797)
Current year of study		
Year 1	26.9% (57)	15.0% (1,258)
Year 2	25.9% (55)	19.2% (1,613)
Year 3 (but not penultimate year)	22.6% (48)	20.1% (1,688)
Year 4 (but not penultimate or final year)	9.0% (19)	9.0% (756)
Penultimate year	11.8% (25)	21.0% (1,759)
Final year	3.8% (8)	15.7% (1,321)
Age		
Median (range)	21 (19–36)	22 (18–51)

### Comparative attrition across specialties

**Fig. 1:**
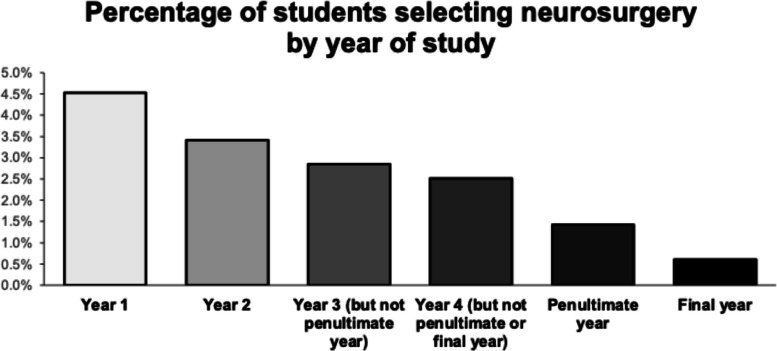
Percentage of students selecting neurosurgery by year of study

A broad decline in specialty preference was observed from first to final year. Neurosurgery demonstrated the steepest reduction, with interest falling from 4.5% of first-year students to 0.6% in final year — the largest attrition of any specialty. Other competitive fields, including cardiology (5.8% to 2.0%) and dermatology (5.1% to 2.0%), also showed marked but less pronounced decreases. In contrast, paediatrics and emergency medicine remained comparatively stable or showed slight increases over time. The full specialty-by-year distribution is provided in the Supplementary Materials, and neurosurgery-specific attrition is illustrated in Fig. 1. As this is a cross-sectional analysis, differences between year groups reflect cohort-level patterns rather than longitudinal tracking of individual students.

### Extracurricular achievements

Achievement types were selected based on alignment with self-scoring criteria used for interview shortlisting across most UK specialty training pathways, including neurosurgery [19, 20]. Neurosurgery aspirants demonstrated higher levels of extracurricular engagement compared to their peers. A greater proportion reported authorship as first authors (5.7% vs. 3.7%) or cited collaborative authors (6.1% vs. 3.8%) on PubMed-indexed publications. Rates of poster (22.2% vs. 20.5%) and oral presentations (18.4% vs. 15.8%) were also slightly higher, though overall involvement in audits or QI projects was lower (15.1% vs. 17.9%).

National leadership roles were more common among neurosurgery aspirants (8.5% vs. 3.4%), as were regional or local roles (20.3% vs. 12.7%). They were also more likely to have received a national or international prize related to medicine (8.0% vs. 4.2%) but less likely to have completed an intercalated or previous degree (25.0% vs. 33.5%). The proportion reporting no extracurricular activities was similar between groups (49.1% vs. 51.1%) (Table 2).

**Table 2:** Extracurricular achievements among neurosurgery aspirants versus the national cohort

Extracurricular activities	Neurosurgery Cohort	National Cohort
	Percentage (n)	Percentage (n)
Research		
First author on a PubMed-indexed publication	5.7% (12)	3.7% (308)
Author on a PubMed-indexed publication	9.9% (21)	10.1% (849)
Cited Collaborative author on a PubMed-indexed publication	6.1% (13)	3.8% (323)
Involvement in an audit/QI project	15.1% (32)	17.9% (1,499)
Poster presentation	22.2% (47)	20.5% (1,717)
Oral presentation	18.4% (39)	15.8% (1,330)
Leadership		
National leadership role	8.5% (18)	3.4% (287)
Regional or local leadership role	20.3% (43)	12.7% (1,067)
Education & examinations		
Medical school examination merit/prize(s)	18.9% (40)	19.3% (1,619)
National or international prize related to Medicine (e.g., essay or conference prizes)	8.0% (17)	4.2% (350)
Intercalated or previous degree	25.0% (53)	33.5% (2,809)
None of the above	49.1% (104)	51.1% (4,293)

### Confidence in securing neurosurgical training posts

Among neurosurgery aspirants, 20.8% reported feeling "confident" or "very confident" in securing a neurosurgical training post, compared to 23.1% in the broader medical student cohort. However, this difference was not statistically significant (OR 0.88, CI [0,62, 1.25], *p* = 0.48) in a multivariable logistic regression model controlling for demographic and educational factors including prior research authorship. Similarly, 39.6% of neurosurgery aspirants were "fairly unconfident" or "not confident at all”, again slightly higher than the 36.5% reported nationally, and this difference was found to be statistically significant (OR = 1.37, CI [1.02, 1.84], *p* = 0.04) in a multivariable logistic regression model, controlling for other demographic and educational predictors including previous research authorship.

Confidence increased with seniority, rising from 22.8% in first-year students to 37.5% in final-year students. While descriptively, students of Mixed ethnicity reported the highest confidence (28.6%), followed by Black (25.0%), Asian (20.9%), and White students (14.7%), these differences were not statistically significant and should therefore be interpreted with caution. Socioeconomic background was associated with higher confidence: students from private schools reported the highest levels (27.0%), followed by those from selective state or grammar schools (21.2%), and comprehensive schools (16.5%). Male gender and prior research authorship were also associated with greater confidence in logistic regression analyses.

### Certainty about specialty choice

A total of 56.6% of neurosurgery aspirants reported being "fairly certain" or "very certain" about their specialty choice, compared with 38.3% of the national cohort. This difference was found to be statistically significant (OR = 2.43, CI [1.82, 3.25], *p* < 0.0001) adjusting for all other predictors in our multivariable logistic model. Uncertainty rates were lower among neurosurgery aspirants, with 21.2% expressing uncertainty compared to 37.6% nationally.

### Knowledge of the neurosurgical training pathway

Among neurosurgery aspirants, 54.7% reported feeling “somewhat” or “very” informed about the neurosurgical training pathway, while only 8.0% felt 'not at all informed. Males were more likely to report feeling informed about the neurosurgical pathway compared to females (67.3% vs. 42.7%). When grouped by ethnicity, those from Mixed ethnicity backgrounds reported the highest levels of knowledge (64.3%), followed by White students (61.8%), Asian students (49.5%), and Black students (43.8%).

### Factors influencing neurosurgery preference

Neurosurgery aspirants identified distinct factors influencing their specialty choice compared to the national cohort. The most commonly cited factor was intellectual challenge, reported by 84.0% of neurosurgery aspirants versus 67.4% nationally, a statistically significant difference (OR = 2.56, CI [1.75, 3.74], *p* < 0.0001) even after adjusting for other predictors including research authorship. Interest in specific conditions was influential for 80.7% of neurosurgery aspirants, compared to 57.1% in the broader cohort (OR = 3.12, CI [2.18, 4.46], *p* < 0.0001). The future outlook of the specialty influenced 75.0% of neurosurgery aspirants, compared to the 70.5% observed nationally, although this difference was not statistically significant (OR 1.34, CI [0.96, 1.87], *p* = 0.09). Research opportunities within the specialty were valued by 73.1% of neurosurgery aspirants, markedly higher than the 37.8% reported by those favouring other specialties (OR = 4.07, CI [2.95, 5.63], *p* < 0.0001).

Conversely, factors highly valued by the wider cohort were less influential among neurosurgery aspirants. Work-life balance was considered important by only 48.6% of neurosurgery aspirants, significantly lower than the 79.0% reported nationally (OR = 0.29, CI [0.22, 0.39], *p* < 0.001). Similarly, levels of stress and pressure at work influenced 37.3% of neurosurgery aspirants, compared to 64.7% nationally (OR = 0.38, CI [0.28, 0.51], *p* < 0.001). Compatibility with family life was influential for 51.9% of neurosurgery aspirants, in contrast to 76.7% of the national cohort (OR = 0.35, CI [0.26, 0.46], *p* < 0.001) (Table 3).

**Table 3:** Factors influencing specialty choice among neurosurgery aspirants versus the national cohort

Factor	Neurosurgery Cohort	National Cohort
	**% Identifying as Influential**	**% Identifying as Influential**
Work and training characteristics		
Work-life balance	48.6%	79.0%
Financial remuneration	62.7%	66.1%
Potential for private practice earnings	49.1%	40.8%
Length of specialty training	26.9%	44.5%
Level of stress and pressure at work	37.3%	64.7%
Level of competition for entry into the specialty	40.6%	49.8%
Future outlook of the specialty	75.0%	70.5%
Training structure (run through vs. uncoupled)	38.7%	39.6%
Number of exams and overall cost of specialty training	34.4%	39.4%
Continuity of care with patients	42.5%	49.2%
Personal and lifestyle considerations		
Compatibility with family life	51.9%	76.7%
Out-of-hours demands (OOH shifts)	42.5%	61.1%
Geographic location preference (e.g., tertiary vs district general vs community)	56.6%	60.3%
Perceived prestige of specialty	42.0%	20.1%
Stereotypes surrounding specialty	24.1%	15.6%
Intellectual and professional growth		
Influence of mentors or role models	60.4%	55.0%
Intellectual challenge	84.0%	67.4%
Research opportunities within specialty	73.1%	37.8%
Use of advanced technology in the specialty	73.1%	31.4%
Use of clinical diagnostic skills vs. investigations	68.4%	53.1%
Interest in specific conditions	80.7%	57.1%
Previous experiences		
Personal experiences of disease	24.5%	27.0%
Pre-clinical positive experiences with the specialty (e.g., lectures, tutorials)	59.0%	54.9%
Past positive interactions with the specialty (e.g., rotations, clinical attachments)	58.5%	72.0%
Demographic preferences		
Preference for working with specific gender groups	5.2%	12.4%
Preference for working with specific age groups (e.g., geriatrics, paediatrics)	16.5%	35.3%
Level of patient interaction	55.7%	77.5%
Diversity of patient interactions	51.9%	64.5%
Gender distribution of doctors in the specialty	20.3%	21.2%

### Medical school distribution

The distribution of neurosurgery aspirants varied across UK medical schools. A complete breakdown can be found in Supplementary Materials.

## Discussion

This study presents the first large-scale, multi-institutional analysis of UK medical students aspiring to neurosurgery, offering unique insights into their demographics, motivations, and preparedness. Unlike previous studies, which have been limited by small sample sizes or self-selected conference cohorts, this analysis draws on a national survey open to all medical students across the UK, with over 8,000 responses. The findings elucidate the profile of neurosurgery aspirants and highlight areas for potential intervention to support recruitment and retention within the specialty.

### Demographic disparities and the neurosurgery pipeline

Our data reveal a distinct gender and ethnic distribution among neurosurgery aspirants, diverging notably from the broader medical student population. While females constitute the majority of UK medical students [21, 22] and of our national cohort, they represent just over half (51.9%) of neurosurgery aspirants, with males disproportionately represented relative to the national cohort (46.2% vs. 30.2%). Neurosurgery was selected by 3.9% of male respondents versus only 1.9% of female respondents. This could suggest that the gender disparity in neurosurgery arises as early as the point of initial career aspiration. These findings align with existing national data from the Society of British Neurological Surgeons, who reported that 23% of neurosurgeons were women. Other studies also show similar gender disparities in other surgical careers [23]. While causality cannot be determined, several factors likely contribute, including low visibility of female neurosurgeons, limited early exposure to the specialty, and persistent perceptions of neurosurgery as incompatible with family life. In our sample, female aspirants were also less likely to express confidence in securing a training post, despite comparable self-reported knowledge of the training pathway, echoing previous research [24, 25]. Addressing these disparities requires deliberate action. Initiatives that demystify neurosurgical training and lifestyle—such as early clinical exposure, transparent discussion of work-life balance, and prominent female role models—may reduce perceived barriers. Additional structural support, including phased return after career breaks and flexible LTFT (less-than-full-time) arrangements, could improve retention and signal to aspiring female surgeons that the specialty is not only accessible, but viable.

### Attrition of interest

Despite longstanding high levels of competition to enter neurosurgical training, medical students’ interest in neurosurgery declines progressively and significantly from 4.5% among first-year students to 0.6% in their final year. This was the highest attrition rate observed across all specialties. Conversely, fields such as otolaryngology, ophthalmology, and anaesthetics saw increases in interest over the same period. This attrition may reflect an initial overestimation of interest among early-year students, possibly influenced by a romanticised perception of neurosurgery as a prestigious and intellectually challenging specialty. However, increased exposure to other specialties and a deeper understanding of the specialty's demands—such as its protracted training, high workload, and burnout risk—likely temper early enthusiasm. Similar trends have been observed in previous investigations, where stress and long training duration were highlighted as key deterrents to pursuing neurosurgery [26].

Limited exposure during medical training may also contribute to this decline. Unlike neurosurgery, which is often underrepresented in medical curricula, specialties such as ophthalmology and anaesthetics benefit from mandatory rotations and a more defined work-life balance, potentially explaining the growth in interest as students progress. This lack of exposure leaves many students graduating without direct neurosurgical experience, hindering informed career decisions and reducing sustained interest [27]. Recent reviews have highlighted the absence of standardised neurosurgical teaching and the inconsistent availability of placements, often due to a perceived lack of curricular priority. National student-led initiatives such as the Neurology and Neurosurgery Interest Group (NANSIG) have attempted to bridge this gap by delivering free, widely accessible teaching programmes, hosting surgical skills workshops, and facilitating mentorship and research opportunities with senior neurosurgeons. Participation in such schemes has been shown to increase student interest in neurosurgery and demystify the training pathway. Expanding these efforts, alongside the introduction of compulsory or structured neurosurgical placements within undergraduate curricula, may help sustain early interest and counter the sharp attrition observed as students progress through medical school.

Beyond curricular gaps, socioeconomic factors may also contribute to attrition. Students from less privileged educational backgrounds were less likely to express confidence in securing a training post, raising the possibility of psychological or structural barriers — such as imposter syndrome, limited access to mentorship, or perceived exclusivity. The relatively limited private practice opportunities in neurosurgery, compared with fields such as orthopaedics or dermatology, may also deter students concerned about financial flexibility. Without financial safety nets, the combination of competitive entry, training intensity, and limited upside may disproportionately discourage otherwise capable candidates. Additionally, the minimum ten-year post-graduation training pathway and perceptions of poor work-life balance may further compound disinterest as students progress [28, 29]. A lack of visible mentors and accessible guidance within the specialty may reinforce these concerns. Given that mentorship is a key driver of specialty selection, its absence may silently redirect talented students elsewhere. It is important that those with genuine aptitude are not deterred by poor visibility, lack of access, or misinformation.

### Confidence, certainty, and knowledge of the neurosurgical pathway

Although neurosurgery aspirants reported high certainty and knowledge of the training pathway, they were significantly more likely to report low confidence, which may reflect a realistic awareness of the specialty’s competitiveness, with a 20:1 applicant-to-post ratio in 2024 [1, 2], and the broader neurosurgical job market where there is a surplus of trained neurosurgeons relative to available consultant posts [30]. This realism may discourage otherwise capable candidates who perceive the pathway as prohibitively selective.

Male neurosurgery aspirants reported higher confidence and certainty compared to their female counterparts. This gender disparity in self-assessment has been documented in other fields and may reflect broader societal patterns of gendered self-perception [31].

Socioeconomic background also appeared to influence confidence. Although the difference in private school attendance did not reach statistical significance, students from private or grammar schools were more likely to report higher confidence in securing a neurosurgical training post. This suggests a structural advantage in perceived self-efficacy, potentially shaped by prior academic environments, access to tailored support, and exposure to competitive career trajectories. Conversely, students from comprehensive schools reported lower confidence levels.

### Extracurricular achievements and the neurosurgical portfolio

Extracurricular achievements among neurosurgery aspirants appeared broadly similar to those of the wider FAST cohort. However, this may reflect the composition of the neurosurgery group, which skewed heavily towards preclinical students. As discussed earlier, early-year medical students appeared more likely to express interest in competitive, high-prestige specialties such as neurosurgery or cardiothoracic surgery, but this interest diminishes sharply with seniority. The limited time available for earlier-year students to develop academic portfolios likely accounts for the absence of marked differences at group level.

This emphasis on extracurricular achievements may create a self-selecting, hyper-competitive environment that prioritises academic credentials over clinical aptitude. This trend aligns with broader patterns of portfolio inflation seen across competitive specialties, where increasing publication output is becoming a prerequisite rather than an exceptional achievement [32]. The growing pressure to publish, sometimes irrespective of quality, risks distorting the intent of academic evaluation and may favour those with greater access, time, or mentorship. Yet in neurosurgery, a strong research foundation remains an asset, given the specialty’s proximity to frontier innovation and scientific inquiry. Efforts to mitigate portfolio inflation must not diminish standards. Selection processes should instead aim to reward depth, originality, and analytical rigour over sheer volume. While academic output should not eclipse clinical skill, neither should it be de-emphasised in the name of superficial fairness.

### Institutional variation in neurosurgical interest

The observed variability in neurosurgical interest across UK medical schools likely reflects a combination of institutional factors. Proximity to tertiary neurosurgical centres, access to research-active departments, and the presence of engaged student surgical societies or neurosurgery interest groups may play a substantial role in fostering early engagement. For instance, institutions with well-established links to neurosurgical units may offer more clinical exposure, academic opportunities, and visible role models, thereby reinforcing aspiration. Conversely, schools with a strong emphasis on primary care or lacking dedicated neurosurgical placements may inadvertently disincentivise interest in the specialty. However, these patterns should be interpreted with caution. Differences may partly reflect sampling artefacts, including response rate variation between institutions or disproportionate representation of specific year groups with naturally higher or lower interest in neurosurgery. Nonetheless, these findings support the argument that institutional culture, opportunity structures, and exposure, not simply individual preference, may shape career intention, and should be considered in future workforce planning.

### International benchmarking and systemic constraints

The constraints observed in UK neurosurgical training should be considered within a broader international context. Across the Anglosphere, neurosurgery consistently comprises less than 1% of all specialty training posts, reflecting a global scarcity of training capacity relative to population size and student interest. In 2023, the UK offered only 16 neurosurgery training posts for a population exceeding 67 million—just 0.13% of all specialty training posts [2]. This translates to roughly 0.16 neurosurgery training posts per 10 million population. By contrast, the United States, with a population of over 330 million, offered 373 neurosurgery training places, representing 0.6% of total training posts and a per capita ratio nearly four times higher than that of the UK [33]. Canada offered 21 training posts in 2023 for a population of approximately 40 million [34]. These international comparisons raise the question of whether UK neurosurgical training capacity remains proportionate to future need. An ageing population, rising burden of neurosurgical disease, and intensifying competition — set against increasing medical school intake — may justify cautious expansion of training posts. However, any such increase must be matched by greater operative exposure and consultant supervision to avoid diluting training quality or undermining standards.

These international comparisons should be interpreted in light of structural differences between healthcare systems, including differences in care delivery models, consultant workload distribution, and the organisation of postgraduate medical training, which may influence training capacity independently of population size.

### Factors influencing specialty choice: the neurosurgical mindset

Neurosurgery aspirants, more so than those aspiring to any other specialty, valued intellectual challenge, research opportunities, and interest in specific conditions. Lifestyle factors, such as work-life balance and family compatibility, were less influential, indicating that neurosurgery appeals to those prioritising intellectual and academic pursuits over lifestyle considerations. The prioritisation of intellectual challenge and research opportunities among neurosurgery aspirants is consistent with global findings [35]. The high level of extracurricular engagement observed among aspirants underscores the importance of fostering research and leadership opportunities during medical education to cultivate interest in neurosurgery. While this focus on intellectual challenge is a strength, it also raises concerns about long-term sustainability. The demanding nature of neurosurgery, combined with lower prioritisation of work-life balance, may increase the risk of burnout, a well-documented issue within the specialty [4, 5, 7, 36]. Early career support, wellness initiatives, and realistic expectation-setting may help mitigate these risks and support long-term retention.

Taken together, these findings suggest that neurosurgery continues to attract a highly motivated and academically oriented cohort, whose commitment appears driven by genuine interest rather than perceived prestige alone.

### Limitations

This study has limitations worth considering. Its cross-sectional design precludes causal inference, and observed differences between year groups reflect cohort-level patterns rather than longitudinal change within individuals. Althpugh the FAST sample size was large, and indeed the largest of its kind conducted in the UK, the subgroup of neurosurgery aspirants was comparatively small (n = 212), particularly in senior years, limiting the reliability of within-group comparisons. Comparisons between neurosurgery aspirants and the wider cohort remain statistically valid; however, subgroup findings should be interpreted with caution due to reduced statistical power. The neurosurgery aspirant cohort may also reflect a self-selecting population with greater academic engagement or confidence, potentially inflating perceived preparedness relative to curricular exposure. All data were self-reported and therefore are subject to recall and response bias. Finally, private school attendance was used as a proxy for socioeconomic background, which — while commonly adopted in UK educational research — may not fully capture the nuance of socioeconomic status. More granular measures such as POLAR quintile, IMD, or parental occupation were not collected to minimise respondent burden and maximise participation and should be incorporated in future work. Recruitment was conducted via national student networks, mailing lists, and social media, constituting a convenience sample; however, participation from all UK medical schools and the large sample size mitigate, though do not eliminate, selection bias.

### Future directions

The literature would benefit from longitudinal studies tracking how interest in neurosurgery evolves throughout medical school and into the postgraduate phase of training. These could help disentangle transient enthusiasm from sustained aspiration and clarify the role of curricular exposure, mentorship, and self-efficacy in shaping career decisions. Qualitative work, particularly involving students who initially considered neurosurgery but later changed paths, would help elucidate the psychological, structural, or cultural barriers underlying attrition. Comparative analysis between those who persist and those who abandon the specialty may uncover modifiable factors in the training environment or support structure. Additionally, research involving current neurosurgical trainees and consultants could provide retrospective insight into motivators, deterrents, and resilience factors. Mapping these findings against medical school experiences may help optimise outreach, selection, and support strategies across the neurosurgical pipeline. Furthermore, in addition to investigating motivation and rationale, it is imperative that the supply of specialty training and consultant posts be expanded to improve the career prospects of neurosurgery aspirants.

## Conclusion

This study provides the first large-scale, multi-institutional snapshot of neurosurgery aspiration among UK medical students. Drawing on data from nearly ten thousand respondents in the national FAST study, it captures the demographic, motivational, and confidence profile of the students who expressed intent to pursue neurosurgery. Aspirants tend to be disproportionately male, privately educated, and from non-White backgrounds, and their interest often wanes over time. Despite high certainty and knowledge of the training pathway, self-confidence of securing a training post remains low, particularly among female students.

Although neurosurgery remains oversubscribed, the observed attrition, socioeconomic skew, and institutional variation may point to systemic issues in exposure, mentorship, and perceived accessibility. The specialty risks losing capable candidates before application. Early and sustained clinical exposure, and visible mentorship are likely to improve recruitment. International comparisons also prompt reflection on whether current UK training capacity aligns with future demand. Any expansion, however, must be carefully matched by operative opportunity and supervision to preserve training quality. Future research should track whether early aspiration predicts eventual entry into training, and examine why interest is lost, with a view to reforming the neurosurgical pipeline.

## Supplementary Information

Supplementary Material 1.

Supplementary Material 2.

Supplementary Material 3.

Supplementary Material 4.

## Data Availability

The datasets generated and/or analysed during the current study are not publicly available, but are available from the corresponding author on reasonable request.

## References

[1] Ferreira T. Escalating competition in NHS: implications for healthcare quality and workforce sustainability. Oxford University Press; 2024. p. 361–5. 10.1093/postmj/qgad131 38204332

[2] NHS England. Competition ratios for 2024. 2025. Available from: https://medical.hee.nhs.uk/medical-training-recruitment/medical-specialty-training/competition-ratios/2024-competition-ratios.

[3] Pascual JSG, Ignacio KHD, Khu KJO. Paving the path to wellness: a systematic review of wellness programs for neurosurgery trainees. World Neurosurgery. 2021;152(206–13):e5. 10.1016/j.wneu.2021.06.047 34146737

[4] Salloum NL, Copley PC, Mancuso-Marcello M, Emelifeonwu J, Kaliaperumal C. Burnout amongst neurosurgical trainees in the UK and Ireland. Acta Neurochir. 2021;163(9):2383–9. 34021783 10.1007/s00701-021-04873-5 PMC8140310

[5] Neal MT, Lyons MK. Burnout and work-life balance in neurosurgery: current state and opportunities. Surg Neurol Int. 2020;11:456. 33408941 10.25259/SNI_736_2020 PMC7771504

[6] Ogbu II, Kaliaperumal C. The future of neurosurgical training in the United Kingdom. World Neurosurg. 2022;168:89–93. 36113712 10.1016/j.wneu.2022.09.038

[7] Jensen TSR, Hakon J, Olsen MH, Gulisano HA, Obbekjær T, Poulsen FR, et al. A national study of burnout, psychosocial work environment, and moral distress among neurosurgical doctors in Denmark. Acta Neurochir. 2025;167(1):53. 39994165 10.1007/s00701-025-06468-w PMC11850451

[8] Hanrahan J, Sideris M, Tsitsopoulos PP, Bimpis A, Pasha T, Whitfield PC, et al. Increasing motivation and engagement in neurosurgery for medical students through practical simulation-based learning. Ann Med Surg. 2018;34:75–9. 10.1016/j.amsu.2018.08.002 PMC6160393 30271592

[9] Whitehouse KJ, Moore AJ. Undergraduate teaching of neurosurgery–what is the current practice in the UK and is there a need for improvement? Br J Neurosurg. 2015;29(6):753–7. 26083138 10.3109/02688697.2015.1054361

[10] Lee KS, Teo M. Letter to the editor regarding" factors influencing medical student interest in a career in neurosurgery". World Neurosurg. 2020;139:655. 32689664 10.1016/j.wneu.2020.03.074

[11] Burford C, Hanrahan J, Ansaripour A, Smith B, Sysum K, Rajwani K, et al. Factors influencing medical student interest in a career in neurosurgery. World neurosurgery. 2019;122:e367–74. 30336295 10.1016/j.wneu.2018.10.056

[12] Brem H, Amundson E. Preparing Hopkins medical students for a career in academic neurosurgery. Surgery. 2003;134(3):414–5. 14555924 10.1067/s0039-6060(03)00120-x

[13] Clark DJ, Kolias AG, Garnett MR, Trivedi RA, Price SJ, Hutchinson PJ. Student-selected components in neurosurgery. Br J Neurosurg. 2016;30(1):4–6. 26610147 10.3109/02688697.2015.1114590

[14] Kolias AG, Trivedi RA. Enhancing the exposure of medical students to neurosurgery. Br J Neurosurg. 2013;27(5):706. 23971721 10.3109/02688697.2013.833166

[15] Akhigbe T, Sattar M. Attitudes and perceptions of medical students toward neurosurgery. World Neurosurg. 2014;81(2):226–8. 23994131 10.1016/j.wneu.2013.08.023

[16] Ferreira T, Collins AM, Handscomb A, French B, Bolton E, Fortescue A, et al. Socioeconomic and demographic predictors of extracurricular achievements among UK medical students (FAST study). BMJ Open. 2025;15(8):e103062. 10.1136/bmjopen-2025-103062 PMC12336483 40780710

[17] Ferreira T, Collins AM, Handscomb A, French B, Bolton E, Fortescue A, et al. Specialty choices among UK medical students: certainty, confidence and key influences—a national survey (FAST study). BMJ Open. 2025;15(8):e103061. 10.1136/bmjopen-2025-103061 PMC12336620 40780717

[18] Ferreira T, Collins AM, French B, Fortescue A, Handscomb A, Plumb E, et al. Factors Affecting Specialty Training Preference Among UK Medical Students (FAST): Protocol for a National Cross-Sectional Survey. JMIR Research Protocols. 2024;13(1):e55155. 39059007 10.2196/55155 PMC11316162

[19] NHS England. 2024 Core Surgical Training Self-Assessment Scoring Guidance for Candidates 2023. Available from: https://medical.hee.nhs.uk/medical-training-recruitment/medical-specialty-training/surgery/core-surgery/core-surgical-training-self-assessment-scoring-guidance-for-candidates.

[20] NHS England. National Neurosurgery ST1 Recruitment: 2025 Shortlisting Matrix - Candidate Version: NHS England,; 2024. Available from: https://www.yorksandhumberdeanery.nhs.uk/sites/default/files/2025_national_neurosurgery_shortlisting_matrix_candidate_version_final.pdf.

[21] General Medical Council. Female representation in medical practice - a journey through time: General Medical Council,; 2025. Available from: https://gmcuk.wordpress.com/2025/03/13/female-representation-in-medical-practice-a-journey-through-time/.

[22] Campbell D. Female doctors outnumber male peers in UK for first time 2025. Available from: https://www.theguardian.com/society/2025/mar/06/female-doctors-outnumber-male-peers-in-uk-for-first-time.

[23] Newman TH, Parry MG, Zakeri R, Pegna V, Nagle A, Bhatti F, et al. Gender diversity in UK surgical specialties: a national observational study. BMJ Open. 2022;12(2):e055516. 10.1136/bmjopen-2021-055516 PMC8968535 35314455

[24] Odonkor MN, Pahwa B, Rincon-Torroella J, Abu-Bonsrah N, Yenokyan G, Dada OE, et al. Effects of Gender and Country of Training on Perceived Access to Opportunities for Neurosurgical Research and Gender-Concordant Mentorship. World neurosurgery. 2025;193:492–510. 39265934 10.1016/j.wneu.2024.09.016 PMC11845301

[25] Bandyopadhyay S, Moudgil-Joshi J, Norton EJ, Haq M, Saunders KE, Collaborative N. Motivations, barriers, and social media: a qualitative study of uptake of women into neurosurgery. Br J Neurosurg. 2022;36(1):19–25. 33215936 10.1080/02688697.2020.1849555

[26] Wani FA, Alanazi KM, Alblwan AS. Factors affecting the choice of neurosurgery as a future career: a cross-sectional study. Cureus. 2024;16(1):e52836. 38406169 10.7759/cureus.52836 PMC10884721

[27] Tiefenbach J, Kaliaperumal C, Demetriades AK. Increasing medical student exposure to neurosurgery: the educational value of special study modules, student selected components, and other undergraduate student projects. Front Surg. 2022;9:840523. 35211505 10.3389/fsurg.2022.840523 PMC8861074

[28] Spurr L, Harris J, Warwick G. So you want to be a brain surgeon? The essential guide to medical careers. Oxford University Press; 2022.

[29] Solomou G, Murphy S, Bandyopadhyay S, Horsfall HL, Mohan M, Chari A, et al. Neurosurgery specialty training in the UK: what you need to know to be shortlisted for an interview. Ann Med Surg (Lond). 2020;57:287–90. 32874557 10.1016/j.amsu.2020.07.047 PMC7452085

[30] Sinha S, McKenna G, Whitfield P, Thomson S, Kitchen N, Surgeons UNSACoTiNatSoBN. Workforce planning in neurosurgery. Br J Neurosurg. 2020;34(1):3–8. 31752554 10.1080/02688697.2019.1692786

[31] Blanch DC, Hall JA, Roter DL, Frankel RM. Medical student gender and issues of confidence. Patient Educ Couns. 2008;72(3):374–81. 18656322 10.1016/j.pec.2008.05.021

[32] Duy PQ, Paranjpe MD, Antwi P, Diab NS, Wang JK, Kim DN-W, et al. Preresidency publication productivity of US neurosurgery interns. World Neurosurg. 2020;137:e291–7. 32014543 10.1016/j.wneu.2020.01.173 PMC7202965

[33] National Resident Matching Program. Results and Data: 2023 Main Residency Match. 2023.

[34] Canadian Resident Matching Service. Quota and applications by discipline: Canadian Resident Matching Service. 2025. Available from: https://www.carms.ca/data-reports/r1-data-reports/r-1-match-interactive-data/.

[35] Adjierteh E, Agbinko-Djogbalar B, Lamptey R, Pekyi-Boateng PK, Adu KO, Abu-Bonsrah N. Factors influencing the medical student’s interest and career choice in neurosurgery. Postgrad Med J Ghana. 2024;13(1):20–6.

[36] Mackel CE, Nelton EB, Reynolds RM, Fox WC, Spiotta AM, Stippler M. A scoping review of burnout in neurosurgery. Neurosurgery. 2021;88(5):942–54. 33471896 10.1093/neuros/nyaa564

