# Supplementary material for: Neurosurgery aspirants in UK medical schools: a national cross-sectional analysis of demographics, motivations, and confidence (FAST study)
Source: BMC Med Educ. 2026 Mar 13;26:649. doi: 10.1186/s12909-026-08845-0 (PMC13101142; doi:10.1186/s12909-026-08845-0)
Supplement: Supplementary file 3 — Supplementary Material 3. [file 12909_2026_8845_MOESM3_ESM.docx]

| **University** | **Count** | **%** |
| --- | --- | --- |
| Anglia Ruskin University School of Medicine | 80 | 1.0% |
| Aston University Medical School | 119 | 1.4% |
| Barts and The London School of Medicine and Dentistry | 225 | 2.7% |
| Brighton and Sussex Medical School | 223 | 2.7% |
| Brunel University London, Brunel Medical School | 78 | 0.9% |
| Cardiff University School of Medicine | 201 | 2.4% |
| Edge Hill University Medical School | 55 | 0.7% |
| Hull York Medical School | 201 | 2.4% |
| Imperial College London Faculty of Medicine | 182 | 2.2% |
| Keele University School of Medicine | 195 | 2.3% |
| Kent and Medway Medical School | 117 | 1.4% |
| King's College London GKT School of Medical Education | 128 | 1.5% |
| Lancaster University Medical School | 130 | 1.5% |
| Newcastle University School of Medical Education | 353 | 4.2% |
| Norwich Medical School | 189 | 2.3% |
| Plymouth University Peninsula Schools of Medicine and Dentistry | 219 | 2.6% |
| Queen's University Belfast School of Medicine | 163 | 1.9% |
| ScotGEM (A combination of St Andrew's and Dundee) | 47 | 0.6% |
| St George's, University of London | 125 | 1.5% |
| Swansea University Medical School | 164 | 2.0% |
| The University of Edinburgh Medical School | 162 | 1.9% |
| Ulster University, School of Medicine | 11 | 0.1% |
| University College London Medical School | 430 | 5.1% |
| University of Aberdeen School of Medicine and Dentistry | 108 | 1.3% |
| University of Birmingham College of Medical and Dental Sciences | 402 | 4.8% |
| University of Bristol Medical School | 321 | 3.8% |
| University of Buckingham Medical School | 133 | 1.6% |
| University of Cambridge School of Clinical Medicine | 545 | 6.5% |
| University of Central Lancashire School of Medicine | 162 | 1.9% |
| University of Dundee School of Medicine | 133 | 1.6% |
| University of Exeter Medical School | 223 | 2.7% |
| University of Glasgow School of Medicine | 268 | 3.2% |
| University of Leeds School of Medicine | 204 | 2.4% |
| University of Leicester Medical School | 273 | 3.3% |
| University of Liverpool School of Medicine | 118 | 1.4% |
| University of Manchester Medical School | 177 | 2.1% |
| University of Nottingham - Lincoln Medical School | 153 | 1.8% |
| University of Nottingham School of Medicine | 165 | 2.0% |
| University of Oxford Medical Sciences Division | 249 | 3.0% |
| University of Sheffield Medical School | 252 | 3.0% |
| University of Southampton School of Medicine | 396 | 4.7% |
| University of St Andrews School of Medicine | 66 | 0.8% |
| University of Sunderland School of Medicine | 71 | 0.8% |
| University of Warwick Medical School | 179 | 2.1% |
|  | 8395 | 100.0% |

Table. Distribution of neurosurgery aspirants by UK medical school
