# Supplementary material for: Neurosurgery aspirants in UK medical schools: a national cross-sectional analysis of demographics, motivations, and confidence (FAST study)
Source: BMC Med Educ. 2026 Mar 13;26:649. doi: 10.1186/s12909-026-08845-0 (PMC13101142; doi:10.1186/s12909-026-08845-0)
Supplement: Supplementary file 4 — Supplementary Material 4. [file 12909_2026_8845_MOESM4_ESM.docx]

| **Specialty** | **Year 1** | **Year 2** | **Year 3 (but not penultimate year)** | **Year 4 (but not penultimate or final year)** | **Penultimate year** | **Final year** |
| --- | --- | --- | --- | --- | --- | --- |
| **General Practice** | 9.1% | 10.7% | 12.4% | 9.4% | 15.7% | 15.3% |
| **Paediatrics** | 11.5% | 10.8% | 9.1% | 10.8% | 12.2% | 10.6% |
| **Anaesthetics** | 3.6% | 4.5% | 4.5% | 7.0% | 7.2% | 9.9% |
| **Emergency Medicine** | 8.8% | 9.1% | 8.6% | 6.5% | 8.5% | 7.4% |
| **Obstetrics & Gynaecology** | 6.4% | 6.8% | 7.3% | 6.1% | 8.4% | 7.3% |
| **Psychiatry** | 5.0% | 4.7% | 5.6% | 5.3% | 5.9% | 4.9% |
| **Trauma and Orthopaedic Surgery** | 6.0% | 6.0% | 5.8% | 4.9% | 5.5% | 4.3% |
| **Acute Internal Medicine** | 0.6% | 1.1% | 1.8% | 1.3% | 1.8% | 2.8% |
| **Plastic Surgery** | 2.8% | 2.0% | 3.1% | 3.8% | 2.4% | 2.5% |
| **Neurology** | 2.3% | 3.2% | 2.8% | 4.4% | 2.3% | 2.5% |
| **Ophthalmology** | 1.7% | 1.9% | 2.1% | 3.4% | 1.9% | 2.5% |
| **Cardiology** | 5.8% | 5.3% | 4.0% | 3.6% | 2.6% | 2.0% |
| **General Surgery** | 3.1% | 3.5% | 3.0% | 3.6% | 3.0% | 2.0% |
| **Otolaryngology (ENT)** | 0.7% | 0.7% | 1.8% | 1.5% | 1.3% | 2.0% |
| **Dermatology** | 5.1% | 3.7% | 4.0% | 2.8% | 2.3% | 2.0% |
| **Radiology** | 1.5% | 1.2% | 1.5% | 1.9% | 1.6% | 1.6% |
| **Clinical oncology** | 1.7% | 1.5% | 1.6% | 1.2% | 0.9% | 1.4% |
| **Neurosurgery** | 4.5% | 3.4% | 2.8% | 2.5% | 1.4% | 0.6% |
| **Cardiothoracic Surgery** | 4.3% | 2.7% | 1.5% | 1.9% | 0.8% | 0.6% |
| **Paediatric Surgery** | 1.7% | 2.2% | 0.9% | 2.2% | 1.1% | 0.5% |
